# Accuracy Analysis of Measuring X-Y-Z Coordinates with Regard to the Investigation of the Tombolo Effect †

**DOI:** 10.3390/s20041167

**Published:** 2020-02-20

**Authors:** Romuald Masnicki, Cezary Specht, Janusz Mindykowski, Paweł Dąbrowski, Mariusz Specht

**Affiliations:** 1 Faculty of Electrical Engineering, Gdynia Maritime University; Morska 81–87, 81-225 Gdynia, Poland; j.mindykowski@we.umg.edu.pl; 2 Faculty of Navigation, Gdynia Maritime University. Morska 81–87, 81-225 Gdynia, Poland; c.specht@wn.umg.edu.pl (C.S.); p.dabrowski@wn.umg.edu.pl (P.D.); m.specht@wn.umg.edu.pl (M.S.)

**Keywords:** marine metrology, geodetic and hydrographic measurements, accuracy, uncertainty evaluation, UAV, TLS methods

## Abstract

Tombolo is a narrow belt connecting the mainland with an island lying near the shore. It is formed as a result of sand and gravel being deposited by sea currents. In consequence, the seabed constantly rises and the shoreline moves towards the sea. This paper deals with accuracy analysis of the undertaken tombolo effect investigation, namely estimation of uncertainty of the measurement results. The aforementioned analysis concerns two methods used for creating a 3D beach model: Firstly, based on geodetic laser scanning (TLS—terrestrial laser scanning) and secondly, using images from unmanned aerial vehicles (UAV). The presented exemplary estimation of uncertainty of the measurement of coordinates X-Y-Z is based on the Polish case study.

## 1. Introduction: Tombolo Effect—Polish Case Study

**Figure 1:**
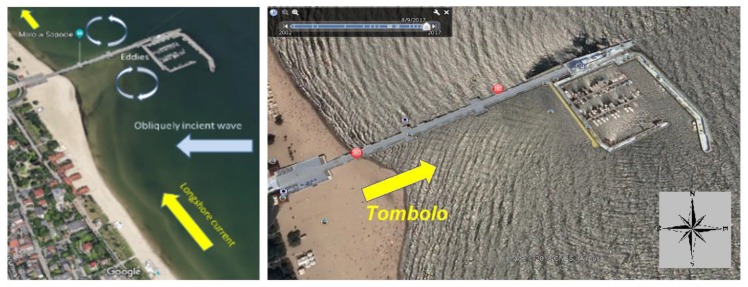
The nature of the phenomenon that causes the formation of the tombolo effect (on the **left**), and the wavy surface of the sea in varying directions resulting from the diffraction of waves of the marina breakwater in Sopot (on the **right**).

**Figure 2:**
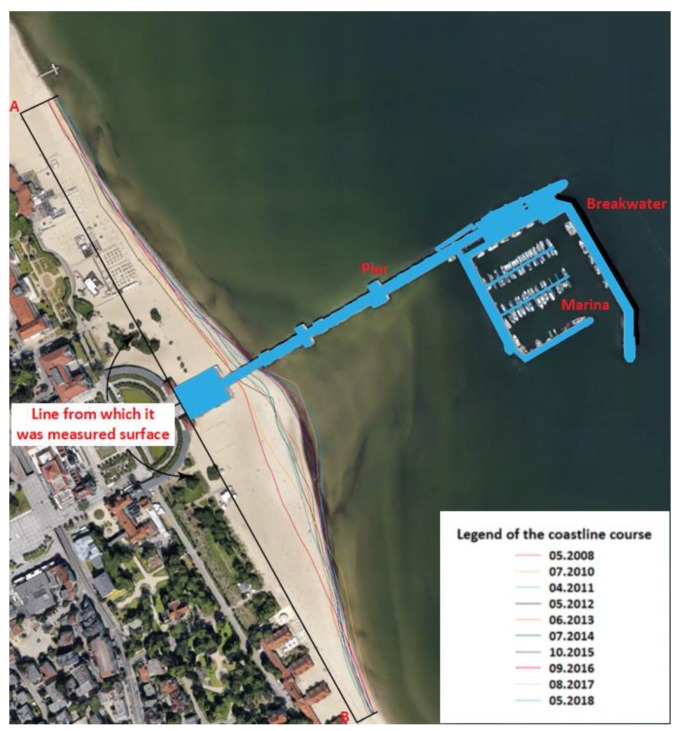
The course of the beach shoreline in Sopot over the years 2008–2018 (with an orthophotomap from 2018). Own study based on [4].

The core of information of this paper is based on a recently published paper [1], describing some metrological aspects of Tombolo effect investigation, namely algorithms applied to estimation of uncertainty of the measurement of X-Y-Z coordinates and metrological properties of the related measurement instrumentation. Sopot is one of the major Polish holiday and spa resorts situated on the Baltic Sea coast. The city has the longest wooden pier in Europe, which is regularly damaged by storms. In October 2009, a violent storm completely destroyed the wooden structure of the pier groyne. The only economically viable method for protecting the pier was to build two breakwaters from the southern and eastern sides. The waterbody bordered between these breakwaters, the pier groyne and head has become a natural marina. In 2010, as a result of discussions of experts and their opinions, it was decided to build a yacht marina in Sopot (three basins, a maximum of 103 vessels: 40 large ones up to 14 m in length, and 63 smaller ones up to 10 m in length) for PLN 72 million. The seemingly right decision is currently becoming a serious problem for the city, as the construction of the marina led to the local stoppage of the sand transport along the coast, which resulted in its accumulation between the marina and the shore and the shift of the coastline towards the sea (approx. 50 m), and initiated the process of the inevitable formation of a peninsula in Sopot. Such an oceanographic phenomenon known as a tombolo [2] is most frequently influenced by the course of beaches and coasts under natural conditions but can also result from human activities, as is the case in Sopot [3]. In the Bay of Gdansk, the strongest surface wind waving is generated from direction N towards E. The waves that reach the beach in Sopot from the E direction hit diagonally against the shore and cause the movement of bottom sediments along the coast. After the marina was built, its breakwater significantly decreased the wave energy, moreover, waves are deflected at its ends (Figure 1), which results in the formation of two vortexes (directed opposite to each other). Consequently, the seafloor between the marina (obstacle) and the shore is elevated upwards, which results in the development of a morphological formation known as a tombolo. It should be stressed that this phenomenon in Sopot is unique in Poland. Figure 2 shows the changes of the beach shoreline near the Sopot pier.

The aforementioned coastline evolution caused by the described phenomenon should be continuously monitored to evaluate the current consequences of the Tombolo effect and to plan the counter measures for protecting investigated pier and neighboring area. With regard to these needs, the paper deals with complex geographical measurement X-Y-Z coordinates using two sources of data, firstly based on geodetic laser scanning (TLS method) and secondly, using images from unmanned aerial vehicles (UAV method). A key point of the undertaken study is focused on accuracy analysis, but first of all on the estimation of uncertainty of carried out measurements. The added value of this paper concerns the authors’ elaboration methodology of accuracy analysis with required data processing for UAV method, being an important completion of the known procedure for TLS method. Additionally, a design and implementation of the complex measurement system for TLS method, completed by case study-based evaluation of uncertainty under real, terrain conditions are presented.

Following this chapter, the rest of the article is structured in this way: In Section 2, a problem to solve is formulated. Section 3 shortly describes the methodology of geodetic and hydrographic measurement used for the studied area of the shore and the seabed, Section 4 presents basic information about applied algorithms and metrological parameters of the related measurement instrumentation, and Section 5, fundamental for this paper, is devoted to the accuracy analysis of the measurement results. The last section is a conclusion of the study.

## 2. Problem to Solve

**Figure 3:**
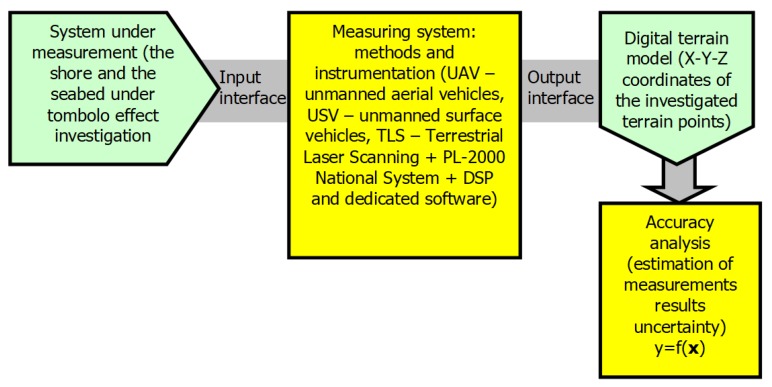
Geodetic and hydrographic measurements of the X-Y-Z coordinates of the investigated terrain points, designations concerning the function y = f(x) are explained in the text.

The paper deals with measurements of the terrain geometry including the shore and seabed under the Tombolo effect investigation. The aim of this research is to determine the X-Y-Z coordinates of investigated terrain points, i.e., continuous cartographic monitoring in this area. Starting from the concept of the meta-model of measurement presented in [5], a flow-chart of performed measurements is shown in Figure 3. The key point of the undertaken measurements is focused on accuracy analysis, understood by the authors as an estimation of uncertainty in relation to carried out measurements. The aforementioned analysis concerns two methods used for creating a 3D beach model: Firstly, based on geodetic laser scanning (TLS—terrestrial laser scanning) and secondly, using images from unmanned aerial vehicles (UAV). Finally, the problem to solve is to assess the accuracy and effectiveness of considered measurement methods under real, terrestrial conditions. 

There is a metrological tradition to distinguish between direct and indirect measurement methods [6], where the first are understood as directly referring to a standard of the relevant measurand, while the second provide measurand estimates computed on the basis of the results obtained by means of some direct measurement methods. In [5], it was noted that this classification has lost its clarity today since the computing resources have been incorporated into the measuring systems, and physical standards have been replaced by their digital representations created during the calibration of these systems. In consequence, the results of almost all today’s measurements are obtained via digital processing of data using mathematical models, which appear in the meta-model of measurement described in [5]. The minimum version of the proposed meta-model of measurement includes such components as mathematical model of system under measurement, mathematical model of data conversion, and mathematical model of data reconstruction. Based on this description, the author of [5] concluded that the propagation of uncertainties through those models is a principal operation underlying the procedures for evaluation of uncertainties. In accordance with this approach, in the vast majority of practically important cases, those models may be decomposed into elementary algebraic structures in the form [5]:

(1) y=f(x)

where $x\equiv {\left[x_{1},x_{2}\ldots \right]}^{T}$ is a vector of variables modeling the sources of uncertainty, *y* is a variable modeling the quantity whose uncertainty is to be evaluated, and *f* is a function describing relations between aforementioned variables. 

The commonly used probabilistic approach is based on the assumption that *x* is a realization of a random vector *x* whose probability density function is *p(x),* and consequently, *y* is a realization of a random variable *y*, whose probability density function is *p(y)* and it results from the propagation of the *x* distribution by the *f* function.

For uncorrelated input quantities the square of the combined standard uncertainty associated with the output estimate *y* is given by:

(2) $$u_{c}^{2}\left(y\right)=\sum_{i=1}^{N}{\left(\frac{\partial f}{\partial x_{i}}\right)}^{2}u^{2}(x_{i})$$

where $\frac{\partial f}{\partial x_{i}}$ are the sensitivity coefficients determined experimentally by measuring the change in *y* produced in a particular *x_i_* while holding the remaining input quantities constant.

For correlated input quantities the combined standard uncertainty associated with the output estimate can be expressed as:

(3) $$u_{c}^{2}\left(y\right)=\overset{N}{\underset{i=1}{\sum }}\overset{N}{\underset{j=1}{\sum }}\frac{\partial f}{\partial x_{i}}\frac{\partial f}{\partial x_{j}}u(x_{i},\;x_{j})=\overset{N}{\underset{i=1}{\sum }}{\left(\frac{\partial f}{\partial x_{i}}\right)}^{2}u^{2}(x_{i})+2\overset{N-1}{\underset{i=1}{\sum }}\overset{N}{\underset{j=i+1}{\sum }}\frac{\partial f}{\partial x_{i}}\frac{\partial f}{\partial x_{j}}u\left(x_{i},\;x_{j}\right)$$

where *x_i_* and *x_j_* are the estimates of X_i_ and X_j_ and *u(x_i_, x_j_)* = *u(x_j_, x_i_)* is the estimated covariance associated with *x_i_* and *x_j_.*

The degree of correlation between *x_i_* and *x_j_* can be estimated by the correlation coefficient described by the formula:

(4) $$r\left(x_{i},x_{j}\right)=\frac{u\left(x_{i},x_{j}\right)}{u\left(x_{i}\right)u\left(x_{j}\right)}$$

where r(x_i_, x_j_) = r(x_j_, x_i_), and −1 ≤ r(x_i_, x_j_) ≤ + 1. 

If the estimates *x_i_* and *x_j_* are independent, *r(x_i_, x_j_)* = 0, and a change in one does not imply an expected change in the other.

In most of the literature, e.g., in [7], the analyses are usually carried out assuming that no temporal or spatial correlation exists between individual laser shots, i.e., each TLS measurement is assumed to be statistically independent. This assumption ignores, for example, the likelihood that similar ranging errors will exist in adjacent laser returns due to observation of common terrain morphology. Although ignoring the correlation between individual 3D point errors will result in more conservative (larger) volume errors, it will be seen that propagated volume errors are already quite small in comparison to the computed volumes even without the inclusion of the mitigating effects of correlation [7]. Moreover, the accuracy estimates can be often determined under a simplifying assumption that the function *f* may be linearized in the small vicinity of the expected value because, as a rule, the evaluation of uncertainty is useful in research practice if this uncertainty is small enough [5].

In the case of considered research, the error components cannot be linked by a functional dependency, and assuming no correlation exists between components, the accuracy of TLS measurements is quite easy to evaluate. The uncertainties associated with this direct-measurement method have their main sources in instrumental inaccuracies and inaccuracies of the reference data. Since the UAV altitude when taking pictures is not exactly determined, the Z coordinate of each point in the terrain model must be reproduced from a series of photos [8,9]. The uncertainty of the results obtained by the UAV method is more difficult to estimate. These measurements are partly indirect measurements (Z coordinate), but the propagation function of their uncertainty is unknown. While the X-Y coordinates of individual points are determined directly on the basis of measurements (by scaling and matching photos), Z coordinates are designated programmatically as a result of the conversion of two-dimensional photos processed into 3D images, accompanied by significant noise effects. An additional component of the Z coordinate inaccuracy is the low precision in determining the height at which the photographs were taken. Thus, accuracy can be estimated by statistical methods and by comparing results with results obtained by another method, e.g., TLS.

## 3. Methodology of Geodetic and Hydrographic Measurements to Illustrate the Studied Area of the Shore and the Seabed

In order to conduct the proposed study, the team of researchers decided to apply and integrate, in measurement terms, two technical solutions whose development began as late as in the second decade of the 21st century, i.e., unmanned aerial vehicles (UAV) [10] and unmanned surface vehicles (USV) [11,12]. They will enable the investigation of the tombolo phenomenon in Sopot in two basic geospatial aspects: 

1. Photogrammetric—designed to assess changes in the beach surface relief and the coastline course based on 3D land modeling using photogrammetric methods (UAV) [13]. The analysis of the phenomenon covers an 800 × 200 m area (beach).

2. Hydrographic—aimed at determining the seafloor relief, the amount of material (sand) accumulated in the vicinity of the pier and the marina, and the level of increase in its volume as a function of time. To this end, acoustic sounding of the waterbody with the dimensions of 800 × 400 m should be regularly carried out. In view of the depths (of less than 1 m) prevailing in this region, it is recommended to use a hydrographic unmanned surface vehicle (USV) [3]. 

During the USV measurements, two independent positioning systems were used. The first system consisting of a low-cost multi-GNSS receiver (u-blox NEO-M8N) with a built-in Fluxgate sensor was used to control the hydrographic unit course in automatic mode, using the PixHawk Cube autopilot. According to its technical data, the accuracy of determining the position of this device is 3–5 m (p = 0.95). The second positioning system was the Trimble R-10 geodetic receiver, working in real time, using the Real Time Satellite Geodetic Network—VRSNet ensuring horizontal accuracy of position determination at the level of 3–5 cm (p = 0.95) [14]. This is the typical accuracy of the majority of such networks working in the world. This receiver was connected to a single beam echo sounder SonarMite BTX (SBES). 

Creating a 3D beach model can be done on the basis of two methods: Using images from unmanned aerial vehicles or making measurements based on geodetic laser scanning [15]. 

**Figure 4:**
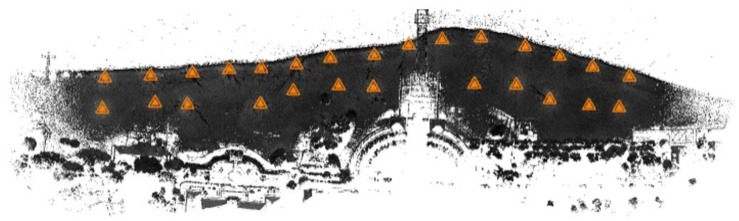
Terrestrial laser scanning (TLS)—the location of laser scanner measurement sites.

In view of the elongated surface nature of the beach under measurement, it was necessary to plan and arrange an appropriate number of sites. The TLS cloud comes from terrestrial laser scanning. The scanner was placed on a number of positions on a geodetic tripod each time the instrument was leveled. The measurement was taken with a Trimble TX8 laser scanner without the photo-taking option. Hence, the obtained point clouds had only colours resulting from the calculated laser beam reflection intensity. To cover the assumed study area, it was necessary to establish 27 sites located at a distance of approx. 60 m from each other (Figure 4). Due to the small number of characteristic objects to be used for the recording of a point cloud in the field at a later time, spherical tags located in the sand in a manner ensuring the stability of their position were used. The tags had to be located in a relatively short distance from the neighboring measurement sites; therefore, they were located halfway between them or in their immediate vicinity. Such an approach ensured that a relatively large set of points on the spherical tag’s surface were obtained during the measurement. This enabled the precise fitting of spheres into the set of points and the determination of their midpoints, which in the recording process were the points of adjustment of particular local point cloud systems.

Photogrammetric flight path was performed using a DJI Mavic Pro drone independently for each adopted part of the area under investigation. The data were recorded using a Pix4D Capture mobile application. During the mission, 621 photographs were taken at an altitude of 60 m above the drone take-off level (the beach). The obtained average GSD coefficient value amounted to 2.25 cm/pix at a camera resolution of 4000 × 3000 effective pixels. The overlap parameter defining the degree of photograph overlapping in transverse and longitudinal directions was determined to be 80%. The main parameters of the camera are: Sensor: 1/2.3” (CMOS), effective pixels: 12.35 M (total pixels: 12.71 M); Lens: FOV 78.8–28 mm (35 mm format equivalent) f/2.2; Distortion: < 1.5%; Focus: From 0.5 m to ∞.

**Figure 5:**
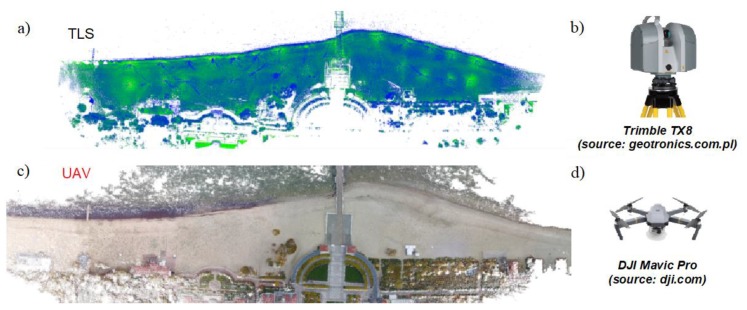
TLS and unmanned aerial vehicles (UAV) methods: a) and c)—point clouds originating from the measurement using two methods, b) and d)—sensing devices, respectively, to TLS and UAV methods.

Generating point clouds from photos requires the use of structure-from-motion (SFM) processing chain [16,17]. A photogrammetric model, i.e., UAV point cloud, was developed using the Pix4d Mapper Pro software which enables photographic data processing and generating three-dimensional models and orthophotomaps. A median of 21,128 nodal points on a single photograph was obtained, which indicates a relatively high number of characteristic points and areas, considering the frequently little diversified coverage of the area surface. A comparative picture of point clouds is illustrated in Figure 5, obtained from measurements using TLS and UAV methods. 

Further parts of this paper contain the metrological analysis of measurements relating to changes in beach surface and shoreline.

## 4. Applied Algorithms and Metrological Properties of the Related Measurement Instrumentation

### 4.1. TLS Method

**Figure 6:**
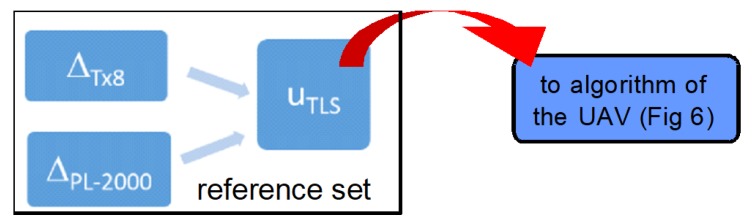
Algorithm for estimating the accuracy of the TLS method, where: ∆_TX8_, ∆_PL-2000_—absolute errors corresponding to the Trimble Tx8 laser scanner and PL-2000 national system, respectively, U_TLS_—uncertainty of the considered method.

The applied laser scanning method concerns the measurement of the X-Y-Z coordinates of the investigated terrain points. This method is referred to here as the TLS method because the measurement was taken with a Trimble Tx8 laser scanner. The analyzed method shows a very good accordance of measurement results with the mandatory system of geographic coordinates. The measurement results obtained in the local system are transferred to the PL-2000 national system. The accuracy of the final data results depends only on the laser instrumental accuracy and accuracy of the reference points location, that is the points that were used to transform the data into the PL-2000 system (Figure 6). 

In the discussed TLS case study, the detailed inaccuracy components of the applied laser scanner measurement come from:

- Inaccuracy of laser Trimble Tx8—1 cm horizontally and 1.5 cm vertically,
- Inaccuracy of satellite receiver R10 used to determine the coordinates of reference points—1 cm horizontally and 1.5 cm vertically,
- Inaccuracy of the PL-2000 system—2 cm horizontally and vertically.

A cloud of TLS points after transformation to the PL-2000 system, including the data collected at the determined area (terrain), can be a reference set (Figure 6) for cloud of points obtained by use of the UAV method in the form of terrain digital photos. A series of photos, processed by the adequate program, enables creating a spatial projection of the investigated area (terrain), in which, besides X-Y coordinates, the Z coordinate is determined in relation to each point of the cloud. On the basis of the reference points coordinates, the obtained local coordinates are transformed into the PL-2000 system. In practice, the sequence of operations realized on the basis of photo-data does not allow achieving a satisfactory level of accuracy of the data in the area covered by the study.

### 4.2. UAV Method

In the UAV method of terrain imaging, the picture processing operation resulted in the generation of a cloud containing 20,666,253 points, which translates into a density of approx. 117 points per m^2^. Numerical data processing was carried out using a high-performance workstation (16 GB RAM, i7-6600U, GTX 1070), running the Windows 10 operating system, and lasted for 3 h 42 min. A list of used software tools can be found in Section 3. The following values of maximum errors for particular coordinates were obtained: 2.83 (X), 4.08 (Y), and 9.81 m (Z). 

In the discussed UAV case study, the detailed inaccuracy components of the applied unmanned aerial vehicle are equal to:

- Camera pixel resolution—2.25 cm,
- Inaccuracy of UAV position: X, Y—a few meters, Z—several meters.

### 4.3. A Proposal on How to Improve UAV Accuracy

It is worth noting that the complexity, as well as the cost of using the UAV method is many times lower for the TLS method. On the other hand, levels of accuracy in UAV measurements are unacceptable. 

To improve the quality of the UAV method measurement, it is proposed to use the TLS data cloud as reference data. These data are obtained in the same area as the data in the UAV data cloud. TLS measurements appear to be reliable compared to available terrain maps, while UAV measurements are burdened with both systematic and random influences. 

**Figure 7:**
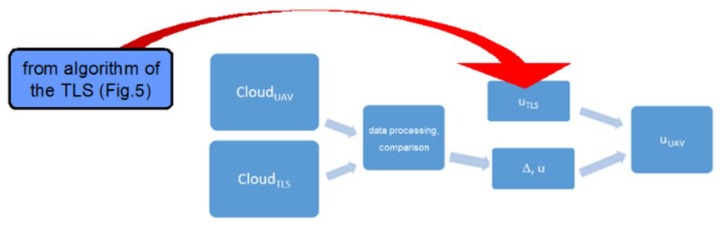
Algorithm of the UAV method accuracy estimation, where: *Cloud_UAV_*, *Cloud_TLS_*—clouds of points related to the methods UAV and TLS, respectively, Δ*, u*—absolute values of occurred errors and related uncertainties, *u_TLS_, u_UAV_*—measurements uncertainties characterizing the methods TLS and UAV, respectively.

Taking the TLS point cloud as a reference data, it is possible to estimate the systematic errors components of the X-Y-Z coordinates in the UAV cloud, as well as their dispersion (noise) in relation to reference values (Figure 7). 

To solve the problem of inaccuracy of UAV measurements, dedicated software is being designed, which is currently in the testing phase. It will allow the determination of the X-Y-Z coordinates uncertainty characteristics collected in large data files obtained using the UAV method.

## 5. Accuracy Analysis of the Measurement Results

### 5.1. Evaluation of Uncertainty

Results of the executed measurements of the terrain geometry are treated as random variables, that is they are subject to statistical rules and the probability calculus is used to assess them. The term “Accuracy analysis” in this paper concerns the estimation of uncertainty in relation to the carried-out measurements of the shore and coastline under investigation. 

The methods for evaluation of uncertainty by statistical tools are tagged in the GUM with the label *type A* evaluation [18,19,20]. Those methods are not applicable if neither statistical information (data) characterizing the vector x nor statistical information (data) characterizing the variable y is available. In the absence of statistical data, the evaluation of the uncertainty of y may be only based on nonstatistical information, such as previously acquired measurement data, experience with or general knowledge of the behavior and properties of relevant materials and instruments, manufacturer’s specifications, data provided in calibration and other certificates or uncertainties assigned to reference data taken from handbooks [18,19,20]. The methods for evaluation of uncertainty by means of nonstatistical tools are also tagged in the GUM with the label *type B* evaluation [18,19,20]. 

A usually applied procedure for evaluating uncertainty of measurement according to [18] includes the following steps:

1. Evaluation of standard uncertainties of input values for *type A* evaluation and/or *type B* evaluation depending on the kind of data,
2. Calculation of sensitivity coefficients *δy/δxi*,
3. Calculation of combined standard uncertainty *u_c_(y),* and finally,
4. Calculation of expanded uncertainty *U = k∙u_c_(y).*

The measurement results obtained with use of the aforementioned methods defined as TLS and UAV, respectively, create the series (clouds) of measurement points. Each of them has different reference in the X-Y-Z coordinates system. In consequence, it is possible to estimate an uncertainty in determining the spatial coordinates of each measurement point in cloud with applying the methodology of *type B* evaluation [18,19,20]. In the case under consideration, the error components cannot be linked by a functional dependency. Assuming no correlation exists between components, *type B* evaluation mainly concerns the estimation of inaccuracy of measurement instrumentation. Its calculation requires determining the absolute error threshold value Δ*X_t_* and then assuming the given function of probability distribution for this error. Then, for a given component, the *type B* standard uncertainty *u_s_(x*) for uniform distribution is expressed as:

(5) $$u_{S}\left(x\right)=\frac{\Delta X_{t}}{\sqrt{3}}$$

where Δ*X_t_*—maximum permissible error threshold value, *u_s_(x)*—standard uncertainty.

Taking into account that estimation of *type B* in the measurement procedure may concern a few inaccuracy components characterized by the standard uncertainties *u_s1_(x), u_s2_(x), . . ., u_sn_(x),* with sensitivity coefficients of 1, we can calculate combined uncertainty *u_c_(x)* as:

(6) $$u_{c}\left(x\right)=\sqrt{u_{s1}^{2}\left(x\right)+u_{s2}^{2}\left(x\right)+\cdots u_{sn}^{2}\left(x\right)}$$

where *u_s1_(x), u_s2_(x), . . ., u_sn_(x)* correspond to all sources of errors in the considered measurement procedure.

In [21], an analysis of the positional errors of terrestrial laser scanning (TLS) data based on 3D data is presented. The 3D point positional accuracy of every point in the space was analyzed among others by the conventional method (modular errors analysis). The point position error is a vector with three Cartesian components, one for each axis X, Y, and Z, and denoted Δ*x*, Δ*y,* and Δ*z*, respectively. The modular error (Δ*m*) is the magnitude equivalent to the square root of the sum of the squares of the previous terms:

(7) $$\Delta m=\sqrt{\Delta x^{2}+\Delta y^{2}+\Delta z^{2}}$$

Such approach is reasonable according to the technical characteristics of the TLS measurements. This is one of the main advantages of the modular error analysis [21]. 

A similar approach is presented in [22] and [23]. The standard deviation of the positional error of any point in the cloud, taken as the representative parameter of positioning quality, is simply given by:

(8) $$\sigma_{p}=\sqrt{\sigma_{x}^{2}+\sigma_{y}^{2}+\sigma_{z}^{2}}$$

where the standard deviation in the X, Y, and Z directions is *σ_x_*, *σ_y_*, and *σ_z_*, respectively.

The same assumptions were used in [24] to assess the error of velocity of a point moving in space as well as in [7] to estimate the snow volume.

Then, for a given component, the *type B* expanded uncertainty *U_B_(x)* is expressed as:

(9) $$U_{B}\left(x\right)=k\cdot u_{c}\left(x\right)$$

where *k*—coverage factor.

Finally, the 3D equivalent expanded uncertainly *U_Be_* of the given point position in the space may be calculated as:

(10) $$U_{Be}\left(X,Y,Z\right)=\sqrt{U_{B}^{2}\left(X\right)+U_{B}^{2}\left(Y\right)+U_{B}^{2}\left(Z\right)}$$

where X, Y, Z—point coordinates, respectively; *U_B_(X*), *U_B_(Y), U_B_(Z)*—coordinate expanded uncertainties of the given point position.

### 5.2. Case Study Based Evaluation of Uncertainty for TLS Method

First of all, the detailed uncertainty components can be analyzed and calculated for the method TLS and related to its measurement instrumentation. Considering known instrumental and method data, the following error components, resulting from previously described experiments (Section 4) are considered: Reference system TLS accuracy—described by standard uncertainty *u_s1_*, satellite receiver R10 maximum errors accuracy—described by standard uncertainty *u_s2_*, PL-2000 system errors threshold values accuracy—described by standard uncertainty *u_s3_*. In consequence, the related combined standard uncertainties *u_c_(X), u_c_(Y),* and *u_c_(Z),* estimated for all three coordinates X,Y, and Z, can be calculated as:

(11) $$u_{C}\left(X\right)=\sqrt{u_{S1}^{2}\left(X\right)+u_{S2}^{2}\left(X\right)+u_{S3}^{2}\left(X\right)}$$

(12) $$u_{C}\left(Y\right)=\sqrt{u_{S1}^{2}\left(Y\right)+u_{S2}^{2}\left(Y\right)+u_{S3}^{2}\left(Y\right)}$$

(13) $$u_{C}\left(Z\right)=\sqrt{u_{S1}^{2}\left(Z\right)+u_{S2}^{2}\left(Z\right)+u_{S3}^{2}\left(Z\right)}$$

The uncertainty budget and related values of standard and combined uncertainties are shown in Table 1 for X or Y coordinates and in Table 2 for Z coordinate.

Based on the literature of the considered issue, all the aforementioned inaccuracy components *u_s1_, u_s2_, u_s3_* are characterized by the uniform probability distribution [18,19,20] and noncorrelated each other, so we can assume the normal distribution of combined uncertainties. In consequence, to calculate the *type B* expanded uncertainty using formula (9), a value of coverage factor *k* = 2 has been chosen. It results from the propagation law of uncertainties [18,19,20] and the analyzed case study condition, when the resultant standard uncertainty is a function of uncorrelated standard uncertainties of three or more quantities with a uniform distribution, then a convolution of three or more uniform distributions approach a normal distribution [18,19,20]. Taking into account aforementioned considerations and using formulas (9) and (10) we can obtain for all coordinates: The expanded uncertainty *U_B_(x)_TLS_* is 2.82 cm (X or Y coordinate) and 3.36 cm (Z coordinate) (p = 95%). Finally, the 3D equivalent expanded uncertainty *U_Be_* (X, Y, Z) = 5.21 cm.

### 5.3. Future Works—Methodology of Accuracy Analysis for UAV Method

The UAV terrain model is easy to create, with much less workload and lower costs than using the TLS method. Since the accuracy of the UAV model is far from expectation, work on improving it has begun. Future works will be focused on carrying out conducting in-depth comparative accuracy analysis of the 3D beach model being created using photogrammetric measurements (derived from UAV), when adopted as a reference model derived from measurements using a laser scanner. The point cloud obtained from the TLS method can be used to assess the accuracy of the UAV method and to correct the systematic errors that accompany it. 

This task is not trivial, bearing in mind the exemplary number of data shown in Section 4.2, it means “the picture processing operation resulted in the generation of a cloud containing 20,666,253 points”. Software being developed for UAV measurement accuracy assessment performs the data analysis in several steps programmed in the algorithm implementing:

- Organizing point clouds: Sorting by X-Y coordinates,
- Random noise filtering,
- Determining standard deviation within selected areas (UAV),
- Determining systematic interactions by comparing UAV and TLS data, correction of point Z coordinates,
- Presenting the results of the assessment and their analysis.

**Figure 8:**
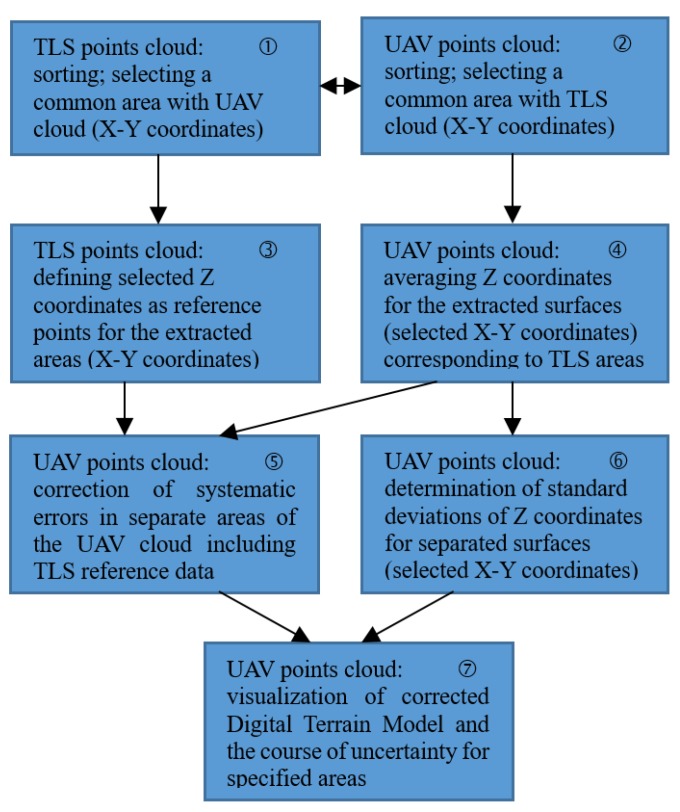
A scheme of actions to be taken to improve the UAV terrain model.

Below, in Figure 8, a block diagram for UAV measurement accuracy assessment is presented and shortly described. 

The terrain model obtained from the raw UAV points cloud is burdened with both systematic and random errors. This is evident in the form of a shift in the Z coordinates of individual terrain points from the reference values and a noise scatter of the Z coordinates of adjacent points. Figure 8 shows the proposed procedure for removing both adverse effects. 

The first step is to make a coordinated arrangement of both point clouds according to the X-Y coordinates ①, ②. The goal is to find a common area for both point sets. Next, operations are carried out in accordance with certain assumptions regarding the resolution of the terrain model in the X-Y coordinate system. As mentioned in Section 4.2, the pixel resolution of camera used in the UAV method is 2.25 cm. This resolution is redundant for terrain modeling for practical reasons. Imaging the considered area according to the sparser grid, e.g., 30 × 30 cm, seems sufficient. For the adopted resolution of terrain imaging, in the TLS cloud a reference point is determined for each of the extracted segments ③, while in the UAV cloud the point cloud filtering [25] is performed, and then the average Z coordinate for points in each of the extracted segments is determined ④. In the next step ⑤, using the reference data from ③, systematic errors are corrected in the UAV points cloud after averaging, obtained in operation ④. In ⑥, the standard deviation of the Z coordinates is calculated for each specified sector. The final operation ⑦ includes both the visualization of the corrected terrain model and the course of systematic errors, as well as the distribution of random errors along the X-Y coordinates.

## 6. Final Conclusions

The coastline around the pier in Sopot is constantly changing. This is mainly caused by the construction of an additional breakwater protecting the pier against storms. It is therefore, necessary to conduct continuous cartographic monitoring in this area. 

The article presents the methodology for conducting this monitoring and the methodology for assessing its accuracy, pointing out: The UAV method (images) is less accurate, but allows the Digital Terrain Model to be made in a relatively short time and with the use of inexpensive equipment; however, the TLS method (laser scanning) is time-consuming, precise, and requires expensive equipment. For this reason, the aim of the undertaken study is to assess the accuracy and effectiveness of both methods under real, terrestrial conditions.

Future works will be focused on carrying out in-depth comparative accuracy analysis of creating a 3D beach model using photogrammetric measurements (derived from UAV), when adopted as a reference model derived from measurements using a laser scanner. The point cloud obtained from the TLS method can be used to assess the accuracy of the UAV method and to correct the systematic errors that accompany it. 

The software being developed using Python environment for UAV measurement accuracy assessment performs the data analysis in several steps programmed in the algorithm implementing: Organizing point clouds with sorting by coordinates, random noise filtering, determining standard deviation within selected areas, determining systematic interactions by comparing UAV and TLS data, correction of point coordinates, presenting the results of the assessment and their analysis.

## Figures and Tables

**Table 1:** Uncertainty budget (X or Y coordinate) of TLS system.

Quantity	Maximum Error	Probability Distribution	Standard Uncertainty
(X,Y)*_Tx8_*	1 cm	uniform	*u_S1_* = 0.58 cm
(X,Y)*_R10_*	1 cm	uniform	*u_S2_* = 0.58 cm
(X,Y)*_PL-2000_*	2 cm	uniform	*u_S3_* = 1.15 cm
*u_TLS_*(X,Y)			*u_c(X,Y)_=* 1.41 cm

**Table 2:** Uncertainty budget (Z coordinate) of TLS system.

Quantity	Maximum Error	Probability Distribution	Standard Uncertainty
(Z)*_Tx8_*	1.5 cm	uniform	*u_s1_* = 0.87 cm
(Z)*_R10_*	1.5 cm	uniform	*u_s2_* = 0.87 cm
(Z)*_PL-2000_*	2 cm	uniform	*u_s3_* = 1.15 cm
*u_TLS_*(Z)			*u_c_*(Z) = 1.68 cm

## Abbreviations

The following abbreviations are used in this manuscript:

CMOS	Complementary Metal-Oxide Semiconductor
FOV	Field of View
GNSS	Global Navigation Satellite Systems
GSD	Ground Sampling Distance
GUM	Guide to the Expression of Uncertainty in Measurement
RAM	Random-Access Memory
TLS	Terrestrial Laser Scanning
UAV	Unmanned Aerial Vehicles
USV	Unmanned Surface Vehicles
**Nomenclature**	
X,Y, Z	point coordinates
*Cloud_UAV_*, *Cloud_TLS_*	clouds of points related to the methods UAV and TLS, respectively
**x**	vector of variables modeling the sources of uncertainty
*y*	variable modeling the quantity whose uncertainty is to be evaluated
Δ*_Tx8_*, Δ*_PL__-2000_*	absolute errors corresponding to the Trimble Tx8 laser scanner and PL-2000 national system, respectively
Δ*X_t_*	maximum permissible error threshold value
*p(x), p(y)*	probability density functions
*u_TLS_, u_UAV_*	measurement uncertainties characterizing the TLS and UAV methods, respectively
*u_s_(x)*	standard uncertainty
*u_c_(x)*	combined standard uncertainty
*U_B_(x)*	*type B* expanded uncertainty
*U_Be_(X,Y, Z)*	3D equivalent expanded uncertainty
Δ*m*	modular error of a vector of the 3D point position
Δ*x*, Δ*y*, Δ*z*	Cartesian components of the point position error
*σ_p_*	the positional error standard deviation of a point in the cloud
*σ_x_, σ_y_, σ_z_*	standard deviations in the X, Y, and Z directions
